# Factors Associated with Failed Trial of Labor after Cesarean, among Women with Twin Gestation—A Multicenter Retrospective Cohort Study

**DOI:** 10.3390/jcm11154256

**Published:** 2022-07-22

**Authors:** Tzuria Peled, Hen Y. Sela, Jordanna Joseph, Tal Martinotti, Sorina Grisaru-Granovsky, Misgav Rottenstreich

**Affiliations:** 1 Department of Obstetrics & Gynecology, Shaare Zedek Medical Center, Faculty of Medicine, Hebrew University of Jerusalem, Jerusalem 91031, Israel; tzuria10@gmail.com (T.P.); hysela@szmc.org.il (H.Y.S.); jordannajoseph@hotmail.com (J.J.); tal.martinotti@gmail.com (T.M.); sorina@szmc.org.il (S.G.-G.); 2 Department of Nursing, Jerusalem College of Technology, Jerusalem 91031, Israel

**Keywords:** cesarean delivery, delivery, trial of labor after cesarean, twin

## Abstract

Objective: Twin trial of labor after a cesarean section (TOLAC) is associated with a lower success rate of vaginal delivery than singleton TOLAC, and a higher rate of adverse outcomes in comparison to an elective repeat cesarean delivery. This study aims to investigate the factors associated with failed TOLAC, among women with twin gestation. Study design: A multicenter retrospective cohort study was undertaken. All women with twin pregnancies attempting a trial of labor after a previous cesarean in two university-affiliated obstetrical centers, between 2005 and 2021 were included. The study population included women with a twin gestation where twin A presented in the vertex position, a single previous low segment transverse section, and those who were eligible for a vaginal delivery. Labor, maternal, and neonatal characteristics were compared. A univariate analysis was undertaken, followed by multivariate analysis (aORs; [95% CI]). Results: A total of 160 women attempting a twin TOLAC were included. Vaginal birth after cesarean was achieved in 86.3% of these cases. Assisted reproductive technology (ART), the lack of oxytocin use for augmentation during labor, the lack of epidural analgesia, and preterm birth before 34, 32, and 28 gestational weeks were all found to be associated with failed TOLAC. In the multivariate analysis, cervical dilation on admission (aOR 0.6 [0.40–0.82], *p* < 0.01), no use of oxytocin (aOR 5.2 [1.36–19.73], *p* = 0.02), gestational age at delivery (aOR 0.8 [0.65–1.00], *p* = 0.047) and lack of epidural analgesia (aOR 4.5 [1.01–20.16], *p* = 0.049), were all found to be significantly associated with failed TOLAC. Conclusion: In the investigated population of women with twins undergoing TOLAC, the use of epidural analgesia, the use of oxytocin and increased cervical dilation to the delivery room are associated with a higher rate of vaginal delivery, and may reduce the risk of repeat cesarean delivery.

## 1. Introduction

Trial of labor after a previous cesarean (TOLAC) is a salient point of discussion in the obstetric literature and clinical setting. Due to the known risks associated with cesarean delivery (CD), including neonatal and maternal morbidities, as well as the increased risk for future obstetrical complications, [1] it is imperative to minimize the rates of repeated CD, more specifically in women who are interested in multiple births in the future. Despite this, TOLAC is not without risk. An unsuccessful TOLAC (one that results in an urgent CD), carries a greater risk of maternal morbidity, including post-partum hemorrhage (PPH), hysterectomy, uterine rupture, operative injury, and greater risks of neonatal morbidity, than a repeat elective cesarean [2,3].

Twin TOLAC has largely been associated with a lower rates of successful vaginal delivery than singleton deliveries [4,5]. Although some studies that have shown similar success rates to singleton TOLAC’s [6,7]. The relatively wide range of success rates is due to the various approaches taken by medical centers in when managing twin TOLAC [5,7]. Despite the associated risks, for the appropriate candidates, twins TOLAC is associated with fewer maternal and neonatal complications compared to twin elective CD [7,8].

Women undergoing twin TOLAC are considered at greater risk for obstetric complications than singleton TOLAC, such as uterine rupture or adverse perinatal outcomes including perinatal mortality [4,5,9]. Conversely, some studies identify the same rates of uterine rupture and other complications in both singleton and twin TOLAC [7]. Studies presenting a higher mortality rate among twin TOLAC may be influenced by prematurity and twin gestation complications. Therefore, when considering the risks of elective CD, twin TOLAC may be considered as a legitimate option [10].

Selecting the correct candidates for twin TOLAC will increase the odds of successful VBAC and decrease the risk of complications. Women with twin gestation can be offered a TOLAC when meeting a specific criterion and following counseling.

The aim of this study is to identify the factors associated with a failed TOLAC, among women with twin gestation.

## 2. Materials and Methods

### 2.1. Study Design

A multicenter retrospective cohort database study was conducted in two university-affiliated obstetrical centers in Jerusalem, Israel: The Shaare Zedek Medical Center (SZMC) and the Bikur Holim Medical Center (BHMC), both serving Jerusalem’s population (>1,200,000 patients). Together these medical centers account for approximately 16% of all deliveries in the state of Israel, with a mean annual volume of 22,000 births. All antepartum and delivery care is covered by the National Health Plan. Data was extracted from the electronic medical record (EMR), which is recorded in real-time at the point of care.

### 2.2. Study Population

The study population comprised of all parturient women with a twin gestation and one previous CD that were found eligible for TOLAC, and gave birth between August 2005 and December 2021 in SZMC or BHMC. Inclusion was limited to women with cephalic presentation of twin A and those who were admitted to the labor ward for a trial of labor. The exclusion criteria included mono-chorionic mono-amniotic twins, antepartum fetal death of one or both fetuses, known genetic or structural anomalies of either twin, home/car delivery, planned CD or an unplanned CD before the trial of labor.

A trial of labor after cesarean may be offered to women with a history of one prior low transverse CD following a discussion of the potential risks, benefits, and alternatives. All women attempting trial of labor after cesarean are managed by resident physicians and supervised by a board-certified obstetrician. Decisions regarding induction or augmentation on labor, operative vaginal deliveries, or emergency CDs are joint decisions. In cases of disagreement about the management, the present board-certified obstetrician is the one who make the decision. Vaginal births are delivered by certified nurse-midwives.

According to our department protocol, induction of labor is suggested at 36–37 gestational weeks for monochorionic-diamniotic (MCDA) twins and 37–39 for dichorionic-diamniotic (DCDA) twins. On admission to the labor ward, fetal heart rates are continuously monitored with a twin electronic fetal monitor. During labor and delivery, a board-certified obstetrician, experienced in performing total breech extraction of the second twin or an emergent CD, is present. Neonates in vertex presentation when there is no indication for vacuum are delivered by certified nurse-midwives. Both neonates are assessed immediately following the birth, by the attending pediatrician [11].

### 2.3. Study Outcomes

Maternal and neonatal characteristics were compared between women who delivered by CD and those who gave birth vaginally. The maternal and neonatal adverse outcomes were also reported.

Maternal outcomes included: early post-partum hemorrhage (PPH), blood products transfusion, grade 3 and 4 perineal tears, uterine rupture, and hysterectomy, among others. Neonatal outcomes included: Apgar scores, neonatal intensive care unit (NICU) admission, neonatal asphyxia, meconium aspiration, jaundice, respiratory distress syndrome (RDS), transient tachypnea of the newborn (TTN), and brachial plexus injury, among others. 

Neonatal discordancy was calculated by the formula: second twin weight − first twin weight in grams. Birth weight difference (BWD), presented in as a percentage, was calculated by the formula: (second twin weight − first twin weight)/second twin weight × 100.

### 2.4. Statistical Analysis

Statistical analysis was carried out using SPSS software (version 25 statistical package: IBM, Armonk, NY, USA). Continuous variables with normal distribution were presented as mean and standard deviation, whereas those without normal distribution were presented as a median with an interquartile range. Comparisons were made using Student’s *t*-test and Mann-Whitney test, respectively. Categorical variables were presented as percentages and comparisons were made by Chi-square and Fisher’s exact test as appropriate. All analyses were two-sided and a *p*-value of <0.05 was considered statistically significant. To assess the independent association between the different risk factors and TOLAC failure (i.e., CD), a multivariable logistic regression model was developed. All variables found to be significantly associated with the CD in univariate analysis were included in the multivariate analyses model. Adjusted Odds Ratios (aOR) and 95% confidence intervals (CI) were computed.

## 3. Results

**Figure 1:**
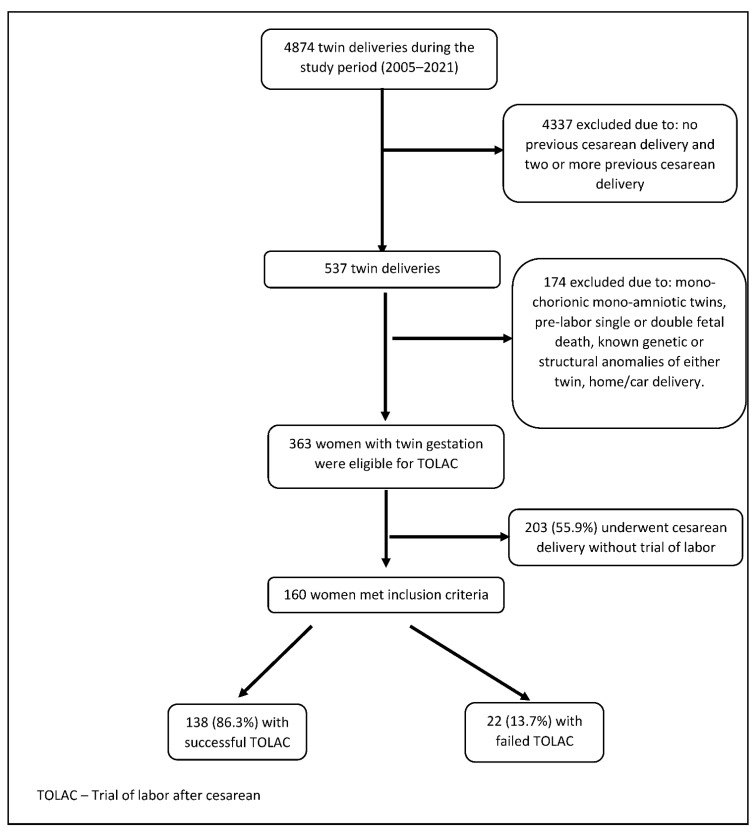
Study flow chart.

During the study period, there were 4874 twin deliveries in SZMC & BHMC, 160 of these women were found to be eligible for inclusion in the study. 138 (86.3%) had successful TOLAC. 22 (13.7%) had failed TOLAC and subsequent CD, of which 2 (9.1%) had a CD for the delivery of the second twin (Figure 1).

Maternal demographic and obstetric characteristics comparing the study and control group are presented in Table 1. Women with failed TOLAC had higher rates of Assisted reproductive technology, and placental abruption, lower rates of premature rupture of membranes, a lower cervical dilation on admission, an earlier gestational age at delivery and similar rates of preterm delivery (<37 gestational weeks). In the investigation of current delivery features, rates of oxytocin augmentation of labor were lower among women with failed TOLAC as well as rates of epidural analgesia use. Other maternal demographic and obstetrics characteristics did not differ significantly between the study groups.

Maternal outcomes are described in Table 2. Women with a failed TOLAC had increased rates of prolonged hospitalization and endometritis. There were no cases of uterine rupture or hysterectomy in the investigated cohort.

Neonatal adverse outcomes were examined for each twin separately (Table 3). In failed TOLAC’s, Twin A was at an increased risk of a lower birth weight, a 1 min Apgar score of <7, NICU admission, prolonged hospitalization, mechanical ventilation, and intracranial hemorrhage. Twin B was found to be at an increased risk of; a large gestational age, a 1 min Apgar score of <7, a 5 min Apgar score of <7, NICU admission, longer hospitalization stay, transient tachypnea of the newborn, and mechanical ventilation.

To assess the independent association between different risk factors and failed TOLAC we fitted a multivariate model (Table 4). The multivariate model revealed that the most important risk factor for failed TOLAC was lower cervical dilation on admission of labor followed by no administration of oxytocin for augmentation of labor. Earlier gestational age at delivery, and lack of epidural analgesia were independently associated with failed TOLAC.

## 4. Discussion

In this multicenter retrospective study of women with twin gestation who underwent a TOLAC, we found a relatively high success rate of TOLAC (86.3%). In cases of failed TOLAC, poorer maternal and neonatal outcomes were identified, in comparison to successful TOLAC’s. Although having low power, we demonstrated few complications associated with failed TOLAC. Specifically, the rate of endometritis was increased, and hospitalization rate was prolonged from 3 to 5 days. The multivariate analysis shows that lower cervical dilation on admission of labor, no administration of oxytocin for augmentation of labor, earlier gestational age at delivery, and a lack of epidural analgesia were the most important factors associated with twin TOLAC failure.

Whilst it is accepted to deliver diamniotic twin pregnancies at 32 0/7 weeks of gestation or later with a vertex presenting fetus, vaginally, [12,13] twin TOLAC remains to be a medical predicament. Studies show that the rates of successful twin TOLAC fluctuate between centers from 57% to 81.5% [5,7,14]. The successful rate of vaginal delivery of twins, particularly when the second twin is non-vertex, is largely dependent on the skill and expertise of obstetricians, the institution’s policy, as well as the maternity population [5], all of which may vary between medical centers. According to our institution’s policy, we enable women with twin gestation and history of CD to have a trial of labor in almost similar criteria as women with singletons. In addition, we encourage women to prefer twin TOLAC on repeat CD in the same way we encourage women to choose singleton TOLAC when the woman is eligible for it. In addition, there is no limitation for women with vertex/non-vertex presentation as all our board-certified obstetrician are qualified to perform total breech extraction.

One meta-analysis identified addressing twin TOLAC presents heterogenous findings. Here, 11 cohort studies were analyzed, most of which were small cohorts, reliant on older data [15]. One study included in the meta-analysis, Ford et al. from 2006, compared 1850 TOLAC cases to 4705 elective CD cases, finding a 45.2% success rate with repeated elective CD having better maternal outcomes [16]. However, this study’s fundamental drawback was that women who were not eligible for TOLAC but were operated on after contractions started were included in the trial of labor group. Moreover, this national study included data from many small obstetrical centers with diverse experiences in vaginal deliveries of twins.

The high success rates of a twin’s TOLAC in this study may be explained by the relatively high volume of vaginal and twin’s deliveries at our medical centers and more importantly, the characteristics of our patients, who have a propensity to plan large families. This factor may also impact patient and physician preferences concerning mode of delivery. Furthermore, the study is based on more recent data than previous studies, and the case’s selection was very strict, by manually reviewing every case, to identify cases with true trials of labor and without other risk factors for vaginal birth failure, to avoid the effect of confounders.

While previous studies report both the success rates and risks associated with TOLAC among women with twin gestation, in comparison to women undergoing a to repeat elective twin CD or to singleton TOLAC, scant literature exists regarding factors associated with successful or failed twin TOLAC [15]. We identified few studies that have considered factors that influence the success or failure of TOLAC in twins, but they were methodology flawed, and their results did not contribute important clinical knowledge.

According to Lopian et al., who investigated 95 women with twin gestation (31 underwent TOLAC), factors that were found to be associated with vaginal delivery failure were maternal age of 35 years or greater, no previous vaginal deliveries or VBACs, and a lower combined neonatal birthweight [5]. This data presents challenges. Firstly, due to the small sample size and secondly, because all factors mentioned are pre-determined and uninterruptible. Alternatively, Hochler et al. studied 235 cases of twin TOLAC and whether the presentation of the second fetus affects the success rates of twin TOLAC, finding no statistical significant differences [14].

The uniqueness of our study is that it does not compare the results to other groups but specifically examines the population group of women who have already been admitted for a twin TOLAC and characterizes the complications and factors leading to a successful vaginal birth. Moreover, the factors identified affecting the success rate of TOLAC, may be intervenable and changeable. Therefore, this data can improve not only the advanced selection process of the appropriate candidate in addition to the management of twin TOLAC during labor, in order to increase the chances of success vaginal delivery.

All the factors that we identified influencing the success or failure of twin TOLAC, have been demonstrated in previous studies investigating singleton TOLAC. Regarding TOLAC in singletons, Trojano et al. reported a greater cervical dilatation on admission to labor was a strong factor determining successful TOLAC [17], with Grisaro et al. demonstrating that epidural analgesia in a TOLAC, is a significant adjunct for successful TOLAC [18]. Literature discussing the impact of oxytocin on singleton TOLAC contradicts what we have found regarding twins. Several studies illustrate that the use of oxytocin increases the chance of TOLAC failure in a singleton [17,18,19], while in our study it increased the chance of TOLAC success in twin pregnancies. It should be noted that at our medical centers, we administer low dose of oxytocin in cases of twins and TOLAC (in singletons and twins), this may be an explanation for the difference. Further research of the use of oxytocin among twins TOLAC is required. 

Fertility treatment was found to be associated with failed TOLAC in univariate analysis but not in multivariate analysis. Previous studies have previously reported that fertility treatment is a risk factor for CD in twin pregnancies [20,21,22]. However, having twins after fertility treatment increased the rate of mainly non-indicated and elective CD, compared to spontaneous twins [21]. Diamant et al. reported that fertility treatment was noted as a risk factor for repeated CD among women with twin TOLAC also in multivariate analysis [23]. The higher rates of CD among women with fertility treatment noted in these and our study could be attributable to greater parental anxiety and obstetric stress surrounding these pregnancies [20].

The uniqueness of the study population, and the fact that our relatively large cohort from two university affiliated obstetrical centers, enabling us to evaluate this uncommon subgroup of women with twin gestation undergoing TOLAC, in a developed country with advanced medical services, is the primary strength of this study. Furthermore, our data was retrieved from real-time updated computerized medical records. Each woman’s entire medical chart, including antepartum, labor and delivery, and postpartum records for each delivery was available, significantly reducing the possibility of bias. In addition, the selective identification of cases reduces the chance of confounders which can impact results; only women admitted to the labor ward for a trial of labor and started trial of labor were included. Women who were eligible for TOLAC but underwent CD before trial of labor, were excluded.

The main drawback of the study is that it is a retrospective study, with the inherent limitations associated with retrospective research designs. In addition, it includes a small sample size due to the uniqueness of this situation. A further limitation is the uniqueness of our population sample that tend to desire large families. This factor may strongly influence both women’s and physician’s preferences concerning mode of delivery. In addition, the findings of the study the relatively higher rates of TOLAC success may attribute our high volume of twin deliveries with all our board-certified obstetrician are qualified to perform breech extraction or cesarean delivery of the second fetus. These factors may affect the generalizability of our findings to other populations.

## 5. Conclusions

In this multicenter study of a population of women undergoing twin TOLAC, we found relatively high success rates of twin success. This high success rate demonstrates that with the right conditions, twins TOLAC is an achievable and legitimate option for women. In this study, we also identified factors that are associated with a successful and failed TOLAC, in twin gestation. Epidural analgesia, the use of oxytocin, and increased cervical dilation to the delivery room were identified to be associated with a higher rate of vaginal delivery and may reduce the risk of repeat cesarean delivery.

These new findings provided an increased understanding to this obstetric situation, which may increase the likelihood of a successful TOLAC and reduce the risk for urgent CD, by better pre-selection candidates for TOLAC, as well as by appropriate interventions during labor. Additional large-scale retrospective and prospective studies from other medical centers are required to strengthen the findings.

## Figures and Tables

**Table 1:** Baseline and labor characteristics of the study population.

	Successful TOLAC N = 138	Failed TOLAC N = 22	*p* Value
Maternal age, years	32.5 ± 5.1	32.3 ± 5.2	0.82
Miscarriages, any	56 (40.6%)	8 (36.4%)	0.71
Miscarriages ≥3	13 (9.4%)	2 (9.1%)	0.96
Gravidity	5.6 ± 3.2	4.5 ± 2	0.14
Parity	4.8 ± 2.6	3.9 ± 1.9	0.11
Fertility Treatments	24 (17.4%)	8 (36.4%)	0.04
Hypertensive disorders of pregnancy	7 (5.1%)	2 (9.1%)	0.45
Smoking	3 (2.4%)	0 (0%)	0.48
Diabetes (pre-gestational & gestational)	10 (7.2%)	1 (4.5%)	0.64
Obesity (BMI ≥ 30)	9 (18%)	2 (22.2%)	0.77
Monochorionic diamniotic	17 (12.6%)	6 (27.3%)	0.07
Anemia (Hb < 11 mg/dL) on admission	21 (15.2%)	4 (18.2%)	0.72
Cervical dilation on admission	4 ± 2.1	2.8 ± 2.2	0.03
Number of vaginal exams	5.4 ± 3.5	4.9 ± 3.7	0.55
Induction of labor	8 (5.8%)	3 (14.3%)	0.16
Oxytocin augmentation of labor	97 (70.3%)	10 (45.5%)	0.02
Meconium-stained amniotic fluid	8 (5.8%)	0 (0%)	0.25
Epidural analgesia	119 (86.2%)	11 (50%)	<0.01
Gestational age at delivery	36.8 ± 1.9	34.9 ± 5	<0.01
Gestational age at delivery <37 week	53 (38.4%)	12 (54.5%)	0.15
Duration of first stage of labor	276.8 ± 273.4	193.5 ± 102.5	0.67
Duration of second stage of labor	391.3 ± 987.7	562.7 ± 273.9	0.57
Placental abruption	4 (2.9%)	3 (13.6%)	0.02
Premature rupture of membranes	24 (17.4%)	0 (0%)	0.03
Inter-twin delivery interval	9.3 ± 10.2	5.8 ± 13	0.15
Twins birthweight discordancy, grams	−32.1 ± 360.3	19.7 ± 394.4	0.54
Birth weight difference, percentage	−2.3 ± 15	−1.7 ± 18.7	0.85
Vertex presentation of second twin	66 (47.8%)	12 (54.5%)	0.56

Data are mean ± standard deviation; number (%); BMI—Body Mass Index, TOLAC—Trial of labor after cesarean.

**Table 2:** Maternal outcomes.

	Successful TOLAC N = 138	Failed TOLAC N = 22	*p* Value
Hospitalization length, days	3.2 ± 1.1	5.4 ± 1.7	<0.01
Prolonged hospitalization	6 (4.3%)	5 (22.7%)	<0.01
Retained placenta/placental fragments	13 (9.4%)	0 (0%)	0.40
Perineal tear grade 3/4	0 (0%)	0 (0%)	N/A
Laceration	2 (1.4%)	0 (0%)	0.57
Episiotomy	11 (8%)	0 (0%)	0.17
Vaginal tear	11 (8%)	0 (0%)	0.17
Maternal ICU admissions	0 (0%)	0 (0%)	N/A
Postpartum hemorrhage	30 (21.7%)	7 (31.8%)	0.30
Hemoglobin drop ≥4 g/dL	10 (7.4%)	2 (9.1%)	0.78
Chorioamnionitis	1 (0.7%)	1 (4.5%)	0.14
Endometritis	2 (1.4%)	3 (13.6%)	<0.01
Blood products transfusion	5 (3.6%)	1 (4.5%)	0.83
Dehiscence of Uterine Scar	0 (0%)	0 (0%)	N/A
Uterine rupture	0 (0%)	0 (0%)	N/A
Hysterectomy	0 (0%)	0 (0%)	N/A
Laparotomy	0 (0%)	0 (0%)	N/A

Data are mean ± standard deviation; number (%); TOLAC—Trial of labor after cesarean; N/A—Non Applicable.

**Table 3:** Neonatal outcomes.

	First Twin			Second Twin		
	Successful TOLAC N = 138	Failed TOLAC N = 22	*p* Value	Successful TOLAC N = 138	Failed TOLAC N = 22	*p* Value
Birthweight	2579.9 ± 479	2299.9 ± 845.4	0.03	2547.1 ± 475.6	2319.6 ± 895	0.07
LGA	24 (17.4%)	3 (13.6%)	0.66	19 (13.8%)	8 (36.4%)	<0.01
SGA	2 (1.4%)	0 (0%)	0.57	7 (5.1%)	1 (4.5%)	0.92
Male gender	67 (48.6%)	11 (50%)	0.90	71 (51.4%)	8 (36.4%)	0.19
1-Minute Apgar score <7	4 (2.9%)	3 (13.6%)	0.02	15 (10.9%)	6 (27.3%)	0.03
5-Minute Apgar score <7	1 (0.7%)	1 (4.5%)	0.14	2 (1.4%)	2 (9.1%)	0.03
NICU admission	33 (23.9%)	10 (45.5%)	0.03	40 (29%)	11 (50%)	0.049
Hospitalization length, days	6 ± 6.7	21 ± 29.7	<0.01	6.7 ± 8.7	25.8 ± 42.3	<0.01
Prolonged hospitalization	38 (27.5%)	12 (54.5%)	0.01	41 (29.7%)	10 (45.5%)	0.14
Meconium aspiration syndrome	0 (0%)	0 (0%)	N/A	0 (0%)	0 (0%)	N/A
Jaundice	9 (6.7%)	3 (13.6%)	0.26	11 (8.4%)	3 (13.6%)	0.43
TTN	7 (5.2%)	1 (4.5%)	0.89	5 (3.8%)	4 (18.2%)	<0.01
Mechanical ventilation	6 (4.5%)	4 (18.2%)	0.01	4 (3.1%)	5 (22.7%)	<0.01
Seizures	1 (0.7%)	0 (0%)	0.69	1 (0.8%)	1 (4.5%)	0.15
Erb’s palsy/fracture of clavicle	0 (0%)	0 (0%)	N/A	0 (0%)	0 (0%)	N/A
Hypoglycemia	16 (11.9%)	2 (9.1%)	0.70	12 (9.2%)	2 (9.1%)	0.99
Sepsis	1 (0.7%)	1 (4.5%)	0.14	0 (0%)	0 (0%)	N/A
Encephalopathy	0 (0%)	0 (0%)	N/A	1 (0.8%)	1 (4.5%)	0.15
Intracranial hemorrhage	1 (0.7%)	3 (13.6%)	<0.01	3 (2.3%)	2 (9.1%)	0.10
Birth asphyxia	1 (0.7%)	1 (4.5%)	0.14	2 (1.5%)	0 (0%)	0.56

Data are mean ± standard deviation; number (%); NICU—Neonatal intensive care unit, TTN—transient tachypnea of the newborn; TOLAC—Trial of labor after cesarean; N/A—Non Applicable.

**Table 4:** Multivariate logistic regression analysis for the association between labor and delivery characteristics and failed TOLAC (Adjusted Odds Ratio).

	*p* Value	aOR	95% CI	
Cervical dilation on admission	<0.01	0.6	0.40	0.82
No oxytocin augmentation of labor	0.02	5.2	1.36	19.73
Gestational age at delivery, weeks	0.047	0.8	0.65	1.00
Lack of epidural analgesia	0.049	4.5	1.01	20.16
Fertility Treatments	0.58	1.5	0.38	5.50

aOR—adjusted Odds Ratio; TOLAC—Trial of labor after cesarean.

## Data Availability

Not applicable.
